# Recent Progress in the Development of Metabolome Databases for Plant Systems Biology

**DOI:** 10.3389/fpls.2013.00073

**Published:** 2013-04-04

**Authors:** Atsushi Fukushima, Miyako Kusano

**Affiliations:** ^1^RIKEN Plant Science CenterYokohama, Kanagawa, Japan; ^2^Department of Genome System Sciences, Graduate School of Nanobioscience, Kihara Institute for Biological ResearchYokohama, Kanagawa, Japan

**Keywords:** metabolomics, metabolite profiling, plant metabolism, hypothesis generation, database

## Abstract

Metabolomics has grown greatly as a functional genomics tool, and has become an invaluable diagnostic tool for biochemical phenotyping of biological systems. Over the past decades, a number of databases involving information related to mass spectra, compound names and structures, statistical/mathematical models and metabolic pathways, and metabolite profile data have been developed. Such databases complement each other and support efficient growth in this area, although the data resources remain scattered across the World Wide Web. Here, we review available metabolome databases and summarize the present status of development of related tools, particularly focusing on the plant metabolome. Data sharing discussed here will pave way for the robust interpretation of metabolomic data and advances in plant systems biology.

## Introduction

Metabolomics, i.e., the measurement of the full suite of metabolites in a living tissue, has expanded greatly over the last decade, especially in the context of biochemical phenotyping. Specifically, in plant science, metabolomic approaches are increasingly used for understanding regulatory networks involved in genotype comparison (Roessner et al., 2001; Weckwerth et al., 2004), measurement of diurnal/circadian rhythms (Urbanczyk-Wochniak et al., 2005; Gibon et al., 2006; Fukushima et al., 2009a; Espinoza et al., 2010), evaluation of genetically modified plants (Catchpole et al., 2005; Baker et al., 2006; Kusano et al., 2011a; Ricroch et al., 2011), uncovering relationships between metabolites associated with carbon and nitrogen metabolism (Stitt and Fernie, 2003; Sato et al., 2008; Kusano et al., 2011b), stress responses (Kaplan et al., 2004; Urano et al., 2009; Caldana et al., 2011; Kusano et al., 2011c; Obata and Fernie, 2012), characterization of many bioresources (Meyer et al., 2007; Rowe et al., 2008; Sulpice et al., 2009), and identifying metabolite quantitative trait loci (mQTLs) (Morreel et al., 2006; Schauer et al., 2006; Lisec et al., 2008; Carreno-Quintero et al., 2012; Matsuda et al., 2012).

**Figure 1:**
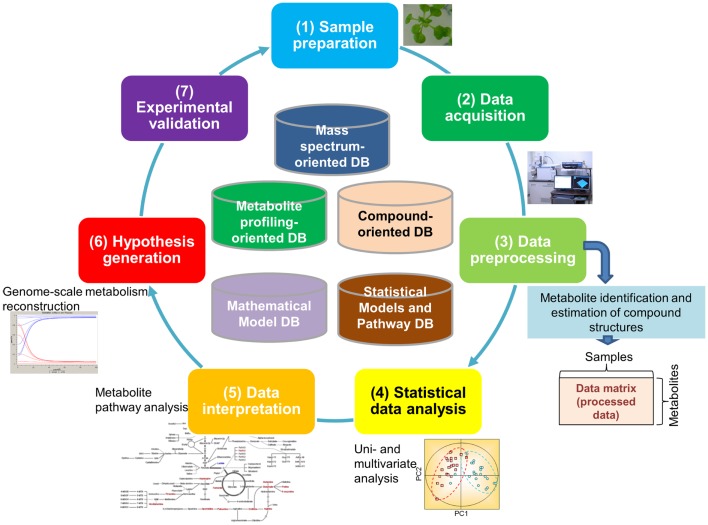
**Major processes generating metabolomic data**. The processes include (1) sample preparation, (2) data acquisition, and (3) data pre-processing. The resultant data go through multi-step downstream processes including (4) statistical data analysis such as principal component analysis, (5) data interpretation such as pathway analysis, (6) generation of testable hypothesis and construction of the model representing the biological phenomenon, and (7) experimental validation of hypothesis and building models.

It is estimated that approximately 200,000 metabolites are produced in the plant kingdom (Fiehn, 2002). There is no single technique suitable for measurement of all metabolites because of the chemical diversity of cellular metabolites and their broad dynamic range, particularly as this pertains to plants (Hall, 2006; Fukushima et al., 2009b; Saito and Matsuda, 2010; Lei et al., 2011; Weckwerth, 2011). Because of this, an array of analytical methods and extraction procedures has been developed for the detection of a broad spectrum of metabolites. Most procedures are based on either mass spectrometry (MS) or nuclear magnetic resonance (NMR). Metabolite data are typically generated through the following processes (Figure 1): (1) Sample preparation, (2) Data acquisition, and (3) Data pre-processing (the first half of the cycle in Figure 1). The resultant data are then subjected to multi-step downstream processes, including (4) Statistical data analysis approaches such as principal component analysis (PCA) and (5) Data interpretation using methods such as pathway analysis, which facilitate (6) The generation of testable hypotheses and the construction of models that best represent the biological phenomenon (the second half of the cycle in Figure 1). Experimental validation (7) of hypotheses and models [(7) in Figure 1] is necessary for closing the systems biology research cycle (Kitano, 2002; Fernie, 2012).

Traditionally, the elucidation of the structure of an unknown natural chemical compound has typically required the study of physicochemical properties, including the accurate mass and chemical shifts in ^1^H- and ^13^C-NMR spectra when the compound was first isolated. In contrast, metabolite identification in metabolomics using gas chromatography–mass spectrometry (GC-MS) and liquid chromatography–mass spectrometry (LC-MS) is not unambiguous. There are two venues to the identification of a chromatographic peak: (1) purification and NMR analysis or (2) interpretation of the spectra yielding a putative structure, followed by synthesizing or buying the compound and spiking. Detectable peaks using GC-MS and LC-MS are thought to be abundant and often authentic standards exist to identify them by spiking. Nonetheless, researchers commonly attempt to provisionally identify these peaks by comparing their mass spectra and/or the retention time or retention indices (RIs) with those present in a database build from the data of authentic standards. To validate the metabolite identification rigorously, the Metabolomics Standard Initiative (MSI)(Fiehn et al., 2007; Sansone et al., 2007) recommends different levels of identification (Sumner et al., 2007). Fernie et al. (2011) have also stated additional practical recommendations for reporting large-scale metabolite data.

It is quite evident that databases cataloging mass spectra and compounds give great support to metabolomic studies (for example, see Tohge and Fernie, 2009; Scalbert et al., 2011). Here, we highlight a wide range of metabolome databases, especially those that are widely used in MS-based metabolite profiling for rapid, but accurate, quantification, and identification of metabolites (Fiehn, 2002). We also discuss further steps to develop future databases facilitating metabolomic analyses and to improve bioinformatics tools in plant systems biology.

## Mass Spectrum-Oriented Information

Since non-targeted metabolite profiling using GC-MS for plant extracts was established in the early 2000s (Fiehn, 2001; Lisec et al., 2006), many software packages and databases for electron impact (EI) mass spectra and RIs of compounds analyzed by GC-MS have been created (Table 1). The NIST/EPA/NIH mass spectral database represents the largest database commercially available for metabolite identification, containing mainly EI mass spectra RIs (Stein, 1999). Recently, the database also stores a set of MS/MS spectra of metabolites, drugs, peptides, and other compounds which are obtained by using ion trap-as well as tandem-MS instruments. The Golm Metabolome Database (GMD) provides GC-EI-MS mass spectral and RI (MSRI) libraries (Kopka et al., 2005; Schauer et al., 2005). It also contains mass spectral tags (MSTs) (Schauer et al., 2005), i.e., MS spectra of putative biological molecules which remain largely unidentified due to the lack of authentic standard compounds. GMD uses both alkanes and fatty acid methyl esters (FAMEs) for RI calculation whereas FiehnLib (Kind et al., 2009), a commercial MSRI library, uses FAMEs rather than alkanes. The Spectral Database for Organic Compounds^1^ (SDBS) includes a wide range of mass spectra for organic compounds, such as polysaccharides. MassBase^2^ is a mass spectral archive for LC-, GC-, and Capillary electrophoresis–MS (CE-MS). SetupX^3^ and BinBase^4^ are a Laboratory Information Management System (LIMS)/database system for automated metabolite annotation and mass spectra, respectively. The Adams library (Adams, 2007), Terpenoids Library^5^, and VocBinBase (Skogerson et al., 2011) are GC-specific MSRI libraries for volatile compounds. The former two are commercially available, while the VocBinBase database is freely available for their provisional identification (Skogerson et al., 2011). For MS data management and data sharing, MetabolomeExpress (Carroll et al., 2010) and MetaboLights (Haug et al., 2012; Steinbeck et al., 2012) were developed. The former is an ftp server that acts as a public data repository and web application for online data pre-processing and meta-analysis of publicly available metabolomic datasets analyzed by GC-MS. The latter is a general metabolomics repository; users can browse publicly available metabolomic datasets, search and see experimental meta-data, and re-use associated data files.

**Table 1:** **Metabolome databases involving mass spectra, compounds, metabolic pathways, metabolite profiles, and statistical/mathematical tools**.

Database	URL	PMID	Notes and contents
**MASS SPECTRUM-ORIENTED**			
AtMetExpress Development	http://prime.psc.riken.jp/lcms/AtMetExpress/	20023150	A phytochemical atlas of *Arabidopsis thaliana* [LC-specific]
BinBase	http://eros.fiehnlab.ucdavis.edu:8080/binbase-compound/	NA	A database system for automated metabolite annotation
Bio-MassBank	http://bio.massbank.jp/	NA	Mass spectra from biological samples [currently, LC-specific]
FihenLib	http://fiehnlab.ucdavis.edu/projects/FiehnLib/index_html	19928838	Mass spectra and RI library based on GC-MS [GC-specific]
GMD@CSB.DB	http://gmd.mpimp-golm.mpg.de/Default.aspx	15613389	GC-MS, retention index, profiles [GC-specific]
MaConDa	http://www.maconda.bham.ac.uk	22954629	A database for mass spectrometry contaminants
MassBank	http://www.massbank.jp/	20623627	Mass spectral database for LC-MS, GC-MS, CE-MS, MALDI-MS, MS2, etc
MassBase	http://webs2.kazusa.or.jp/massbase/	NA	A mass spectral archive for LC-MS, GC-MS, and CE-MS.
MetaboLights	http://www.ebi.ac.uk/metabolights/	23060735	A database for metabolomics data and meta-data.
METLIN	http://metlin.scripps.edu	16404815	Mass spectral database for LC-MS, MS2
MetaboSearch	http://omics.georgetown.edu/MetaboSearch.html	22768229	A software tool for metabolite identification
MS/MS spectral tag (MS2T) viewer	http://prime.psc.riken.jp/lcms/ms2tview/ms2tview.html	18939963	MS2 collections based on LC-MS [LC-specific]
NIST	http://www.sisweb.com/software/ms/nist.htm	NA	Mass spectra based on GC-MS, LC-MS, MS2, etc
ResPect	http://spectra.psc.riken.jp/	22867903	MSn spectrum collection of literature [LC-specific]
SDBS	http://riodb01.ibase.aist.go.jp/sdbs/cgi-bin/cre_index.cgi?lang = eng	NA	Spectral database for organic compounds
SetupX	http://fiehnlab.ucdavis.edu/projects/FiehnLib/:8080/m1/	NA	A management system of mass spectrometric data
The glycan mass spectral database (GMDB)	http://riodb.ibase.aist.go.jp/rcmg/glycodb/Ms_ResultSearch	16053281	Mass spectral database for glycan [MALDI-specific]
The MetabolomeExpress	https://www.metabolome-express.org/	20626915	Online data repository for GC-MS-based metabolomics [GC-specific]
Adams library (4th Edition)		ISBN 978-1-932633-21-4	2,205 MSRI library for plant essential oils [GC-specific]
Terpenoids and related constituents of essential oils	http://massfinder.com/wiki/Terpenoids_Library	NA	>2,400 MSRI library for industry and research in the areas of essential oils [GC-specific]
VocBinBase	http://vocbinbase.fiehnlab.ucdavis.edu/	21816034	MSRI library for volatile compounds in GC-MS [GC-specific]
**COMPOUND-ORIENTED**			
BKM-react		21824409	A biochemical knowledge database
CAS	http://www.cas.org/	NA	The world’s authority for chemical information
ChEBI	http://www.ebi.ac.uk/chebi/	17932057	similar to PubChem
Chemspider	http://www.chemspider.com/	NA	A database for chemical structure
KEGG COMPOUND	http://www.genome.jp/kegg/compound/	22080510	A database for small compounds
KNApSAcK	http://kanaya.naist.jp/KNApSAcK/	22123792	A comprehensive species-metabolite relationship database
LipidBank	http://lipidbank.jp/	NA	A database for natural lipids
LipidMaps	http://www.lipidmaps.org/	17584797	A database for lipid species
Metabolomics.jp	http://metabolomics.jp/	18822113	A wiki database for metabolomics
MetRxn: a knowledgebase of metabolites and reactions spanning metabolic models and databases	http://metrxn.che.psu.edu/	22233419	A web-based database for metabolites and reactions
Plant metabolome database (PMDB)	http://www.sastra.edu/scbt/pmdb/	NA	Annotated database for metabolites in plants
PubChem	http://pubchem.ncbi.nlm.nih.gov	19498078	A database of small organic compounds
The MMD data	http://dbkgroup.org/MMD/	19562197	A database for endogenous and exogenous metabolites
**PATHWAY-ORIENTED**			
AraCyc	http://arabidopsis.org/biocyc/	15888675	Metabolic pathways for *Arabidopsis*
AraPath	http://bioinformatics.sdstate.edu/arapath/	22760305	A knowledgebas for molecular pathways in *Arabidopsis*
BioCyc	http://biocyc.org/	22102576	A collection of pathway/genome databases
IPAD	http://bioinfo.hsc.unt.edu/ipad/	23046449	A pathway analysis database
iPath	http://pathways.embl.de	21546551	A web application for the analysis and visualization of biological pathways
KaPPA-View	http://kpv.kazusa.or.jp/en/	21097783	A web-based database for analyzing omics data
MapMan	http://mapman.gabipd.org/	19389052	A stand-alone tool for analyzing omics data
MetaCrop	http://metacrop.ipk-gatersleben.de	22086948	Metabolic pathways for crops
MetaCyc	http://metacyc.org/	22102576	Metabolic pathways for multi-organisms
MetPA	http://metpa.metabolomics.ca	20628077	A web application for analyzing and visualizing metabolomic data
MetScape	http://metscape.ncibi.org/	22135418	A Cytoscape plugin for visualizing metabolomic data
Paintomics	http://www.paintomics.org	21098431	A web application for the visualization of metabolomic data
Pathos	http://motif.gla.ac.uk/Pathos/	22002696	A web-based database for the storage and analysis of metabolomic data
Pathvisio	http://www.pathvisio.org	18817533	A tool for visualizing biological pathways
PlantCyc	http://www.plantcyc.org/	NA	Metabolic pathways for plants
ProMetra	http://www.cebitec.uni-bielefeld.de/groups/brf/software/prometra_info/	19698148	A viewer for multiple omics data
SMPDB	http://www.smpdb.ca	19948758	A database for small molecule pathways
UniPathway	http://www.unipathway.org/	22102589	A manually curated database for metabolic pathways
VANTED	http://vanted.ipk-gatersleben.de/	16519817	A stand-alone tool for mapping omics data into metabolic pathways
**METABOLITE-PROFILING-ORIENTED**			
*Plants*			
ARMeC	http://www.armec.org/	20003623	A database for ESI-MS-based metabolomics including mainly potato
Chloroplast 2010 Project	http://bioinfo.bch.msu.edu/2010_LIMS	21224340	Metabolite profiles of > 10000 SALK lines
GMD@CSB.DB: the Golm metabolome database	http://gmd.mpimp-golm.mpg.de/Default.aspx	15613389	A metabolome database for plants
KOMIC Market	http://webs2.kazusa.or.jp/komics/	NA	A database for mass spectrometry-based metabolomics
McGill Metabolome Database	http://metabolomics.mcgill.ca/	NA	A metabolome database for crops
Medicinal plant metabolomics resource	http://metnetdb.org/mpmr_public/	doi:10.3390/metabo2041031	A metabolome database for medicinal plants
MeKO@PRIMe	http://prime.psc.riken.jp/meko/	NA	A web-portal for visualizing metabolomic data of Arabdiopsis
Moto DB (Metabolome tomato database)	http://appliedbioinformatics.wur.nl/moto/	16896233	A metabolic database for tomato based on LC-MS
Plantmetabolomics.org	http://www.plantmetabolomics.org	22080512	A web-based database for analyzing and sharing metabolomic data of Arabdiopsis
SoyMetDB: the soybean metabolome database	http://soymetdb.org/	NA	A web-based database for soybean metabolomics
*Animals*			
HMDB	http://www.hmdb.ca/	18953024	Human metabolome database
MMMDB	http://mmmdb.iab.keio.ac.jp	22139941	Mouse multiple tissue metabolome database
SMDB	http://www.serummetabolome.ca/	21359215	Serum Metabolome database
*Bacteria*			
ECMDB	http://www.ecmdb.ca/	23109553	Ecoli metabolome database
YMDB	http://www.ymdb.ca/	22064855	Yeast metabolome database
**TOOLS**			
Chemical translation service (CTS)	http://cts.fiehnlab.ucdavis.edu	20829444	A web tool for translation of chemical information
IMPaLA	http://impala.molgen.mpg.de/	21483477	A web tool for over-representation and enrichment analysis
MBRole	http://csbg.cnb.csic.es/mbrole/	21208985	A web application to perform various types of enrichment analyses
Metab2MeSH: annotating compounds with medical subject headings	http://metab2mesh.ncibi.org/	22492643	A web application for annotating compounds with MeSH
MetaboAnalyst	http://www.metaboanalyst.ca/	22553367	A web application to analyze metabolomic data [multiple functions]
MetaGeneAlyse	http://metagenealyse.mpimp-golm.mpg.de/	14630670	A web application for analyzing omics data [multiple functions]
MetaMapp	http://metamapp.fiehnlab.ucdavis.edu	22591066	A web application to generate network graph using metabolomics data
metaP-Server	http://metabolomics.helmholtz-muenchen.de/metap2/	20936179	A web application to analyze metabolomic data
MetiTree	http://www.MetiTree.nl	22851531	A database for mass spectra of small molecules
MetMask	http://metmask.sourceforge.net	20426876	Integration tool for chemical identifiers
MPEA	http://ekhidna.biocenter.helsinki.fi/poxo/mpea/	21551139	Metabolite pathway enrichment analysis
MSEA	http://www.msea.ca/	20457745	A web application to perform various types of enrichment analyses
MS-MS fragment viewer	http://webs2.kazusa.or.jp/msmsfragmentviewer/	NA	A database for FT-MS-based metabolomics
SMPDB	http://www.smpdb.ca/	19948758	Small molecule pathway database
MeltDB	http://meltdb.cebitec.uni-bielefeld.de	18765459	A web-based system for data analysis and the management of metabolomics [multiple functions]
**GENOME-SCALE METABOLIC MODELS**			
AraGEM	http://web.aibn.uq.edu.au/cssb/resources/Genomes.html	20044452	A genome-scale metabolic reconstrucion in *Arabidopsis*
C4GEM	http://web.aibn.uq.edu.au/cssb/resources/Genomes.html	20974891	A genome-scale metabolic reconstrucion in C4 plants
Poolman’s model	http://www.plantphysiol.org/content/suppl/2009/10/08/pp.109.141267.DC1/141267Poolman_etal_Supl.zip	19755544	A genome-scale metabolic reconstrucion in *Arabidopsis*
Radrich’s model	http://www.biomedcentral.com/1752-0509/4/114/additional	20712863	A genome-scale metabolic reconstrucion in *Arabidopsis*
Mintz-Oron model	http://www.cs.technion.ac.il/~tomersh/methods.html	22184215	A tissu-specific genome-scale metabolic reconstrucion in *Arabidopsis*
BioModel database	http://www.ebi.ac.uk/compneur-srv/biomodels-main/	20587024	A database for mathematical modesl of biological pathways
BiGG	http://bigg.ucsd.edu/	20426874	A high-quality curated database for genome-scale metabolic reconstruction
The model SEED	http://www.theseed.org/models/	20802497	A high-throughput generation system for genome-scale metabolic model

It has been demonstrated that metabolite profiling using LC-MS has the potential to reveal secondary metabolites produced by plants, but most of the detected peaks in LC-MS profile data are largely unknown (Moco et al., 2006; De Vos et al., 2007; Iijima et al., 2008; Matsuda et al., 2010). Compared to EI, LC-MS ionization methods such as electrospray ionization (ESI) does hardly fragment the molecular ions. Even if authentic standards do not exist, putative metabolite identification can be done via MS/MS fragmentation and recording of accurate masses using ultra-high resolution MS such as Fourier-transform ion cyclotron resonance mass spectrometry (FT-ICR-MS) (Lenz et al., 2004; Nakabayashi et al., 2013). Different collision energy/analytical conditions cause different fragment patterns in mass spectra and should also be noted. Several databases have been developed for the sharing of ESI mass spectral information. METLIN stores high-resolution MS/MS spectra at four different collision energies (Tautenhahn et al., 2012). MassBank is a publicly available database of ESI-MS/MS spectra of authentic metabolite standards obtained under five collision energies as well as EI spectra (Horai et al., 2010). Bio-MassBank catalogs those obtained from biological samples^6^. In analogy with the MST spectra obtained from GC-MS data, the MS/MS spectral tag (MS2T) of detectable metabolites using LC-ESI-quadrupole-time-of-flight/MS is also available (Matsuda et al., 2009, 2010; Sakurai et al., 2013). This library contains MS2T obtained from species such as *Arabidopsis thaliana*, rice, soybean, and wheat. Compatible LC-MS settings with those used for the MS2T recording can be used for annotating detected peaks. Based on literature surveys, a plant-specific MS/MS spectra database was constructed by the same group (Sawada et al., 2012). MetaboSearch can be used to simultaneously retrieve mass-based metabolite data from multiple metabolite databases (Zhou et al., 2012). This contains tools to query and/or comprehensively analyze LC-MS-based metabolomic data. Together, these mass spectral databases make an important contribution to metabolite identification and also facilitate the development of bioinformatic tools (e.g., mining unknown metabolites) in metabolomics.

## Compound-Oriented Information and Structure Characterization

### Compound databases

There are also compound databases (Table 1), such as Chemical Abstract Service (CAS)^7^. CAS is the oldest database of chemical information (e.g., journal abstracts); substances in the CAS registry database are each assigned a unique ID number. The PubChem database in NCBI (Wang et al., 2009), ChEBI (Degtyarenko et al., 2008), and ChemSpider (Pence and Williams, 2010) are freely available and can be used to retrieve chemical structures of small molecules. Well-curated chemical information including compounds and pathways are available in KEGG database (Kanehisa et al., 2012), KNApSAcK database (Afendi et al., 2012), and Metabolomics.jp^8^ (Arita and Suwa, 2008). The Plant Metabolome Database (PMDB) is a freely available database of secondary metabolites in plants (Udayakumar et al., 2012). For bioactive lipids, LipidBank (Watanabe et al., 2000) and LipidMap (Fahy et al., 2007) are available. The Manchester Metabolomics Database (MMD) has been developed to simultaneously utilize genome-scale data from the Human Metabolome Database (HMDB)(Wishart et al., 2009), KEGG, and LipidMaps. Other well-organized databases of biochemical knowledge are also available, such as BKM-react (Lang et al., 2011) and MetRxn (Kumar et al., 2012).

### Structural characterization

One of the known bottlenecks in metabolomics is in the identification process of unknown metabolites, which can be classified as either “known unknowns” or “unknown unknowns” (Wishart, 2009). The former corresponds to a metabolite that has been previously detected but has not yet been identified, while the latter corresponds to a truly novel metabolite that has never been formally identified. Schymanski et al. (2012) have shown that consensus structure elucidation using a combination of GC-EI-MS, structure generation, and physicochemical properties calculated from unknown compounds may be applicable to the characterization of unknown metabolites. Kumari et al. (2011) evaluated a novel *de novo* workflow for the annotation of unknown metabolites using accurate mass data, PubChem queries, RI matching, and structure constraints. To predict elemental compositions from accurate mass data collected from high-resolution mass spectrometers, “Seven Golden Rules” (Kind and Fiehn, 2007) and MFSearcher^9^ are available. Krumsiek et al. (2012) demonstrated that the integration of metabolite profiling with genome-wide association studies (GWAS) on metabolic quantitative traits is very useful for deriving biochemical pathways for unknown metabolites. In addition, several groups have attempted to classify unidentified MSTs using supervised machine learning approaches, including decision tree (Hummel et al., 2010) and soft independent modeling of class analogies (SIMCA)(Tsugawa et al., 2011). For structural characterization, there are recent powerful approaches by comparing mass spectral fragmentation trees (Rasche et al., 2011; Hufsky et al., 2012; Rojas-Cherto et al., 2012)(see also the review by Xiao et al., 2012). To evaluate whether detected peaks are biochemically produced by organisms, an *in vivo* ^13^C-labeling system has been used with ^13^CO_2_ in metabolite profiling using both GC-MS (Huege et al., 2007) and LC-MS (Giavalisco et al., 2009). The method allows for the rejection of non-biological peaks and improved annotation of elemental composition. Because artificial biological gradients developed by Redestig et al. (2011) can evaluate actual concentration differences of metabolite peaks detected in two different types of samples (e.g., leaves and fruits), this allows to filter out all unavoidable artifacts in MST/MS2T data. Such a method will make it possible to reject analytical artifacts and prioritize unknown candidate metabolites for further characterization.

## Statistical Models, Pathway Information, and Data Interpretation

### Uni- and multivariate analysis

To perform extensive data analysis such as PCA, MetaGeneAlyse (Daub et al., 2003) and MetaboAnalyst (Xia et al., 2012) are available. Conceptually, these are very similar web-based applications for the analysis of high-throughput omics data. MetaGeneAlyse implements standard normalization/clustering methods, e.g., k-means, and independent component analysis (ICA). MetaboAnalyst provides many statistical methods, including *t*-tests, partial least square discriminant analysis (PLSDA), pathway enrichment analysis, and additional machine learning methods. Please note that several tools and databases presented in this review have multiple functions. Furthermore, several web-based applications for metabolomic data are available (Table 1), such as MetaMapp (Barupal et al., 2012), metaP-Server (Kastenmuller et al., 2011), MeltDB (Neuweger et al., 2008), and MetiTree (Rojas-Cherto et al., 2012). They cover multiple steps from data pre-processing to biological interpretation.

### Metabolite pathway analysis

Metabolite data, which contain information about metabolite name and changes of metabolite levels/relationships, can be described in pathways or networks. For example, the nodes represent metabolites and the edges represent biochemical reactions. Well-curated database for metabolic pathways in plants are available, such as KEGG (Kanehisa et al., 2012) and AraCyc (Zhang et al., 2005). MetaCrop stores well-curated information for 60 major metabolic pathways in eight crop plants, as well as *Arabidopsis* (Schreiber et al., 2012). UniPathway (Morgat et al., 2012) and SMPDB (Frolkis et al., 2010) also provide well-curated information about metabolic pathways. Tools involving pathway analysis and enrichment analysis are also available, such as AraPath (Lai et al., 2012), Kappa-view (Tokimatsu et al., 2005; Sakurai et al., 2011), and MapMan (Usadel et al., 2009) (Table 1). For detailed information about these tools, see the excellent review by Chagoyen and Pazos (2012).

## Mathematical Model Information and Other Tool

### Genome-scale metabolism reconstruction

Over the past few decades, a significant number of metabolic reconstructions have been performed in many organisms, for example, SEED servers (Aziz et al., 2012). Currently, several genome-scale metabolic models in plants are available for evaluating metabolic behavior based on the alteration of metabolic pathways (Table 1) (Collakova et al., 2012; De Oliveira Dal’molin and Nielsen, 2012; Seaver et al., 2012). Poolman et al. (2009) constructed such a metabolism model in *Arabidopsis* to characterize possible flux behaviors using flux balance analysis (FBA) (Orth et al., 2010;Sweetlove and Ratcliffe, 2011). Instead of using metabolic flux analysis (MFA)(for example, see the reviews by Libourel and Shachar-Hill, 2008; Allen et al., 2009), this analysis can predict steady-state flux distribution by using a linear programing. AraGEM is also another metabolic reconstruction of *Arabidopsis* metabolism (De Oliveira Dal’molin et al., 2010). Radrich et al. (2010) semi-automatically integrated multiple databases involving metabolic pathways to reconstruct *Arabidopsis* metabolism. A compartmentalized, reconstructed metabolic model of *Arabidopsis* is also currently available (Mintz-Oron et al., 2012). Combinations of theoretical and experimental approaches will pave the way for robust interpretation of metabolomic data and practical metabolic engineering in plants.

### Tools for metabolite identifiers

Managing compound identifiers in metabolomic data analysis is important. MSI also proposed the use of database identifiers for peer-reviewed papers, for example, the most common compound identifiers, including CAS, KEGG COMPOUND, CHEBI, and HMDB. The Chemical Translation Service (CTS) (Wohlgemuth et al., 2010) and MetMask (Redestig et al., 2010) are a conversion tool for chemical identifiers (Table 1). The former is a web-based tool for performing batch conversions of compound identifiers, while the latter is a stand-alone command line program for integrating the most common compound identifiers. Metab2MeSH (Sartor et al., 2012) is a web application for annotating compounds with Medical Subject Headings (MeSH), which is a controlled vocabulary. Controlled vocabulary means well defined index term is used for indexing journal articles. Metab2MeSH links from metabolites to the biomedical research literature, PubChem, and HMDB. These tools in this subsection are helpful for reporting metabolomic data.

## Metabolite-Profiling-Oriented Information

In addition to mass spectrum and compound databases, several metabolite-profiling databases have also been developed in the past few years (Table 1). Among these, PlantMetabolomics.org (Bais et al., 2010, 2012; Quanbeck et al., 2012) and Medicinal Plant Metabolomics Resource (MPMR) (Wurtele et al., 2012) are one of the most important databases. These contain metabolomic information for >140 *Arabidopsis* mutants and 14 medicinal plants based on MS data from multiple laboratories (Bais et al., 2012). Their profiling broadly covers a wide range of metabolites relating to amino and fatty acids, organic acids, phytosterols, isoprenoids, lipids, and secondary metabolites. PlantMetabolomics.org and MPMR also provide multiple data analysis tools including data normalization and visualization. Using these tools investigators can generate testable hypotheses with respect to gene functions in *Arabidopsis* (Quanbeck et al., 2012). Another example is Chloroplast 2010, which contains data related to large-scale phenotypic screening of *Arabidopsis* chloroplast mutants (Lu et al., 2011), based on assays of amino acids and fatty acids in leaves and seeds using GC-MS and LC-MS (Gu et al., 2007; Bell et al., 2012). Recently, we constructed the MeKO database^10^ (Fukushima et al., submitted), which is similar in concept to PlantMetabolomics.org. MeKO contains metabolomic information on 50 *Arabidopsis* mutants, including plants with uncharacterized gene functions. The website also provides MSI-compliant data, experimental meta-data, and the results of statistical data analyses such as differential accumulation compared with wild-type plants (Columbia ecotype). These databases are very useful for functional genomics and make it possible to develop additional bioinformatic tools for pre-processing of metabolomics raw data, extraction of biologically meaningful mass spectra, and reduction/correction of unwanted variation in large-scale metabolomic data.

## Conclusion

In this review, we have highlighted an extensive list of databases that incorporate both MS-based metabolomics, as well as data analysis tools. Clearly, a small, but significant, number of integrated databases, including the full annotation of metabolites, metabolite profiling, and data analysis tools are emerging, such as PlantMetabolomics.org (Quanbeck et al., 2012). In addition to those for plants, metabolome databases for bacteria and animals also exist (see Table 1). Increases in metabolomic data sharing and the improvement of technological capabilities, such as database integration, are likely to play important roles in the future development of plant metabolomics, and facilitate advances plant systems biology.

## Conflict of Interest Statement

The authors declare that the research was conducted in the absence of any commercial or financial relationships that could be construed as a potential conflict of interest.

## Footnotes

^1^http://riodb01.ibase.aist.go.jp/sdbs/

^2^http://webs2.kazusa.or.jp/massbase/

^3^http://code.google.com/p/setupx/

^4^http://sourceforge.net/projects/binbase/

^5^http://massfinder.com/wiki/Terpenoids_Library

^6^http://bio.massbank.jp/

^7^http://www.cas.org/

^8^http://metabolomics.jp/

^9^http://webs2.kazusa.or.jp/mfsearcher/index.html

^10^http://prime.psc.riken.jp/meko
